# Efficient Wideband Spectrum Sensing with Maximal Spectral Efficiency for LEO Mobile Satellite Systems

**DOI:** 10.3390/s17010193

**Published:** 2017-01-21

**Authors:** Feilong Li, Zhiqiang Li, Guangxia Li, Feihong Dong, Wei Zhang

**Affiliations:** College of Communication Engineering, PLA University of Science and Technology, 88 Houbiaoying Rd., Nanjing 210007, China; 15895970727@163.com (F.L.); uuulzq@163.com (Z.L.); dfh_sinlab@163.com (F.D.); zev@msn.com (W.Z.)

**Keywords:** collaborative sensing, LEO mobile satellite systems, compressed detection, spectral efficiency, hard fusion scheme, scheduling strategy

## Abstract

The usable satellite spectrum is becoming scarce due to static spectrum allocation policies. Cognitive radio approaches have already demonstrated their potential towards spectral efficiency for providing more spectrum access opportunities to secondary user (SU) with sufficient protection to licensed primary user (PU). Hence, recent scientific literature has been focused on the tradeoff between spectrum reuse and PU protection within narrowband spectrum sensing (SS) in terrestrial wireless sensing networks. However, those narrowband SS techniques investigated in the context of terrestrial CR may not be applicable for detecting wideband satellite signals. In this paper, we mainly investigate the problem of joint designing sensing time and hard fusion scheme to maximize SU spectral efficiency in the scenario of low earth orbit (LEO) mobile satellite services based on wideband spectrum sensing. Compressed detection model is established to prove that there indeed exists one optimal sensing time achieving maximal spectral efficiency. Moreover, we propose novel wideband cooperative spectrum sensing (CSS) framework where each SU reporting duration can be utilized for its following SU sensing. The sensing performance benefits from the novel CSS framework because the equivalent sensing time is extended by making full use of reporting slot. Furthermore, in respect of time-varying channel, the spatiotemporal CSS (ST-CSS) is presented to attain space and time diversity gain simultaneously under hard decision fusion rule. Computer simulations show that the optimal sensing settings algorithm of joint optimization of sensing time, hard fusion rule and scheduling strategy achieves significant improvement in spectral efficiency. Additionally, the novel ST-CSS scheme performs much higher spectral efficiency than that of general CSS framework.

## 1. Introduction

Currently, mobile satellite systems (MSSs), adopting Geostationary (GEO) and Non-Geostationary (NGEO) orbits such as Low-altitude Earth Orbits (LEO) and Medium-altitude Earth Orbits (MEO), have attained a renewed R&D interest and market opportunities with extensive application in providing communication services to fixed or mobile users for a vast range of sectors (e.g., land-mobile, aeronautical, maritime, military and disaster relief, etc.), especially in the scenario of terrestrial communication infrastructure congested or gusty paralysis.

Each orbit has some desirable properties as well as a set of drawbacks for efficient service delivery. Due to the concerns regarding the ease of use of large-sized terminals equipped with large antennas and power supplies as handsets, as well as the need for direct radio communications between the user located anywhere around the world, it leads to the idea of using low earth orbit (LEO) satellites at a height between 500 km and 2000 km of altitude rather than the geostationary orbit. Besides, in the case of delay sensitive application, LEO-based MSSs have a typical end-to-end propagation delay of 20–25 ms, which is comparable to the delay of a terrestrial link. On the other hand, considering the mobility issue and the number of LEO satellites required for global coverage, network management becomes more complex and more costly. According to the orbit types, Paolo Chini summarized some basic features of the operational GEO based MSSs and non-GEO based MSSs (i.e., the GEO-based systems of Inmarsat BGAN (Broadband Global Area Network), Thuraya, ACeS (Asia Cellular System), Eutelsat & SES Astra (Solaris Mobile, Pairs, France), Hispasat, MSV (Mobile Satellite Ventures) and TerreStar (Reston, VA, USA); the LEO-based systems of Iridium, Globalstar and Orbcom; MEO-based systems of ICO (Intermediate Circular Orbit)). Details of the satellite orbit, user link frequency bands, physical layer, multiple access, satellite features and ISL can be found in [1,2,3].

However, these systems are spectrum limited due to the static spectrum allocation policies at present. Frequency bands for MSSs are assigned at the World Radiocommunication Conferences (WRCs), which is organized by International Telecommunication Union-Radiocommunication sector (ITU-R), periodically. Although the rapid growth demands for the increasing wide band and data services have promoted the application of Ku/Ka band for MSSs [4], almost all of the operational MSSs regard L/S band as the preferential frequency resources since they permit small onboard antennas due to better foliage penetration and smaller impact of atmospheric affects [5,6]. According to the final documentation of WRCs held in Geneva, Switzerland, 2012 (WRC-12) [7], the allocation of L/S band for mobile satellite services promulgated by ITU Radio Regulation (ITU-RR) is described in Table 1. In order to reuse frequency and alleviate interference, ITU-RR divides the earth into three regions: Regions I, II and III.

The statistical results of Table 1 used by the incumbent systems shows that the scarcity of L/S frequency resources makes it impossible for another planned global or even regional mobile satellite system to seek for a completely pure L/S band, which has attained pervasive attention in those countries who are not in possession of such independently controllable system. In fact, satellite frequency of interest may be not used at a particular geographic location or at a particular time. Thus, exploring efficient spectrum sharing techniques to enhance spectral efficiency in future MSSs without interfering to the operational services has become an important research challenge.

Cognitive radio (CR) proposed by Joseph Mitola is identified as the promising technology to improve the utilization efficiency of the available spectrum [8,9]. As the core function of CR, spectrum sensing enables the secondary user (SU) to adapt to the detected underutilization spectrum without causing interference to primary user (PU) [10,11]. Thus, in terms of discovering the vacant spectrum, CR will provide a competitive approach to obtain frequency for MSSs autonomously. Recently, the application of CR in hybrid/integrated Satellite-Terrestrial communication systems has received important attention lately in the research community. In 2012, Biglieri et al. [12] summarized research papers devoted to the application of CR to satellite communications from 2007 to 2012, with special attention to the implementation of the spectrum sensing technique; Hoyhtya et al. [13] introduced three application scenarios involving satellite acts PU or SU within the terrestrial system and defined the operational limits for interference management in the aforementioned scenarios regarding spectrum sensing. Based on the foregoing, it can be concluded that the application of CR techniques to satellite communication systems has obvious potential to improve spectral efficiency. Concretely, Dimc et al. [14] performed spectrum sensing to find the signal of GSM Thuraya mobile satellite system for both uplink and downlink communication in the frequency ranges 1626.5–1660.5 MHz and 1525–1559 MHz, respectively. Clark et al. [15] considered a real-world scenario involving spectrum sharing between terrestrial mobile wireless and meteorological satellite services in the band of 1695–1710 MHz with the total 15 MHz bandwidth. Hoyhtya [16] focused on the secondary use of the satellite spectrum in the band of 2.17–2.2 GHz by a terrestrial system accompanied by 30 MHz bandwidth. Khan et al. [17] presented cyclostationary analysis of real time satellite signals and its detection technique within a bandwidth of 4 MHz, in which the signal of interest is scanned and captured. These studies focused on the spectrum sharing within narrow band in the scenario of single satellite system. Furthermore, in 2015, Maleki et al. [18] investigated the potential of applying CR techniques in the scenario of Ka band multibeam satellite communications to increase the spectrum opportunities for future generations of satellite networks without interfering with the operation of incumbent services, in which the spectrum opportunities can potentially amount to 2.4 GHz of bandwidth in the downlink and 2 GHz of bandwidth in the uplink for fixed satellite services. However, those explorations of CR applied in satellite communication system from the latest scientific literature face the problem of sensing technology implementation in wideband signal detection.

Actually, wideband sensing techniques are required for detecting wideband satellite signals. The most representative solution is based on filter banks to actualize spectrum sensing in each narrowband at a time. However, this approach requires a large number of RF components and introduces large latency [19,20]. Besides, current analog-to-digital hardware technology faces tremendous challenge posed by conventional spectral estimation method, which operates equal or greater than the Nyquist sampling rate. As the efficient method to achieve real-time spectrum estimation of wideband signal at a sub-Nyquist sampling rate, compressed sensing (CS) combined sampling with compression operations has obtained substantial research efforts. The compressed sampling theory points out that any sparse signal can be compressed and sampled simultaneously [21]. Preliminary work has been done to exploit CS for wideband spectrum sensing based upon signal sparsity in frequency domain. Tian et al. [22] firstly carried out the work to take advantage of CS theory to acquire wideband signals using sub-Nyquist sampling rate. Recently, Duarte et al. [23] presented a structured compressed sensing. Leus et al. [24] proposed a power spectrum blind sampling (PSBS) algorithm trying to reconstruct power spectrum. While the CS literature has intensely focused on problems in signal reconstruction or approximation typically relying on the assumption of sparsity, it is frequently necessary that signals are acquired only for the purpose of making detection in many signal processing applications without reconstructing the signal [25]. In terms of the application of SS in satellite communications due to the spectrum scarcity, only when the wideband signal of primary system is found absent can the SU utilize the spectrum for transmission. In this context, wideband spectrum sensing is equivalent to wideband signal detection. Hence, CS-based wideband signal detection approach, which places no assumptions on the signals being sparse or compressible, provides more spectrum access opportunities for SU with sufficient protection to PU.

The sensing performance of a single node may degrade in wireless channels for several reasons such as the hidden node problem, shadowing, multipath fading, and interference/noise uncertainty. To address these issues, various collaborative spectrum sensing (CSS) schemes among multiple secondary users have been proposed [10,26,27,28,29,30], in which several nodes collaborate with each other to enhance the overall sensing performance. According to the frame structure of periodic spectrum sensing (PSS) [31,32], the sensing time has a direct impact on the sensing performance, such as the probabilities of detection and false alarm, which also affects the data transmission of SU because it determines transmission duration when frame length is fixed. Therefore, there exists a tradeoff between sensing time and transmission duration. The optimal sensing time varies with different optimization objectives. Peh et al. [33] proposed joint optimization of the sensing time and the parameter of CSS scheme to maximize the throughput of the SU. Shi et al. [34] considered a joint design of energy efficient sensing and transmission duration for CR system. Hu et al. [35] investigated the efficient spectrum sensing time with the optimization of transmission delay. Lee et al. [36] developed a theoretical framework to optimize the sensing parameters, in such a way as to maximize the sensing efficiency subject to interference avoidance constraints. Additionally, Min et al. [26] proposed a weighted bandwidth-based cooperative spectrum sensing scheme for satellite communication systems to detect PU in time throughout the whole frame at the cost of degraded spectrum utilization efficiency. To the best of our knowledge, most of the academic and industrial activities are concerning CR in terrestrial wireless communication network for the scenario of narrowband cooperative spectrum sensing. The metrics of spectral efficiency deserves further investigation and discussion, since it becomes particularly important for LEO MSSs due to the scarcity of frequency resources.

This paper mainly investigates the problem of joint designing the compressed sensing time and the parameters (i.e., hard decision fusion threshold, scheduling strategy and number of cooperative SU) of CSS scheme to maximize the spectral efficiency of SU, subject to adequate protection to PU. The main contributions of this paper are as follows. Firstly, we establish the wideband spectrum sensing model based on compressed detection. After compressed sensing, each SU makes local decision (i.e., “1” for presence, “0” for absence) and reports to the fusion center (FC), where counting fusion rule is employed to reach a global decision based on the sensing results. It is proven that SU spectral efficiency is unimodal convex function of compressed sensing time τ. Moreover, inspired by the previous general CSS frame structure, we propose a novel wideband cooperative spectrum sensing (CSS) framework in which each SU reporting duration can be utilized for its following SU sensing. The sensing performance benefits from the novel CSS frame structure because the equivalent sensing time is extended by making full use of reporting time. Additionally, the spatiotemporal CSS (ST-CSS) scheme is designed to achieve space and time diversity gain simultaneously for time-varying channel without introducing additional time overhead. Finally, via computer simulations, it is shown that the proposed CSS framework with optimal settings of sensing time and fusion scheme significantly improves SU spectral efficiency.

The rest of this paper is organized as follows. Section 2 describes the system model. Then, Section 3 introduces the novel cooperative spectrum sensing framework. In Section 4, the optimal settings for the novel ST-CSS with counting fusion rule under sufficient protection to PU is presented. Extensive simulation results are provided in Section 5 to validate the performance of the proposed scheme. Finally, conclusions are drawn in Section 6.

## 2. System Model

**Figure 1:**
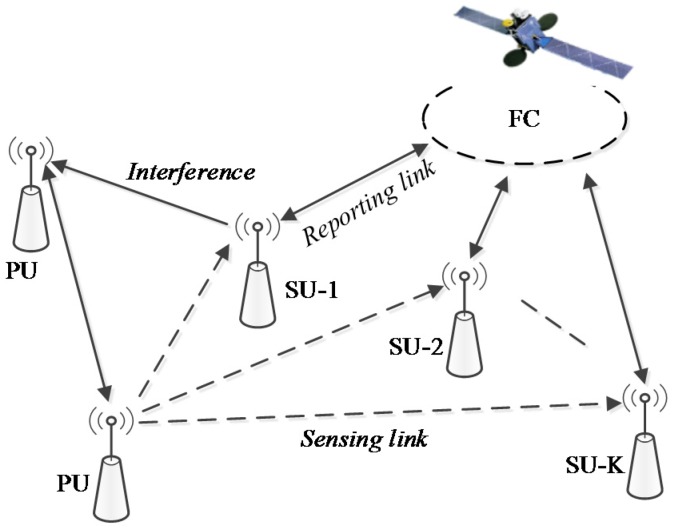
The system model of the CSS (cooperative spectrum sensing) for LEO (low earth orbit) mobile satellite services.

It is observed from the scenarios described in [12,13] that satellites can be provided on a secondary basis, and the primary services can be either terrestrial or satellite-based. The architecture and system model of CSS for LEO mobile satellite services is established as depicted in Figure 1. We consider the CR network consisted of K SUs (i.e., earth stations or sensors) and one LEO satellite (i.e., FC), which are taken as secondary system. Besides, the primary system is composed of PU licensed in the incumbent satellite services or terrestrial networks operating in the same spectrum, such as 3G mobile telephony, LTE, WiMax and WiFi. We define two types of links that we call sensing link (i.e., from PU to SU) and reporting link (i.e., from SU to FC). In this system model, there are broadly three steps: first, each SU implements local sensing to independently detect the presence or absence of PU via sensing link; second, SU reports the detection result to FC through reporting link; and third, FC combines the results with hard counting fusion rule [35,37] to make a global decision about the state of primary system and then reports back to SU. Only when the primary system is detected to be idle can the SU use the licensed frequency for transmission. Once any of PU activity is found, the secondary system must fleetly vacate the spectrum to avoid interference to PU.

We assume that all the SUs in the coverage of satellite can sense the PU. The signals received at SU may come from terrestrial or satellite-based primary users spreading over a wide band. The employed CS-based approach has been developed to detect PU using sub-Nyquist rate samples to reduce the burden on the ADC in wideband spectrum sensing (WSS). In the compressed settings, the problem of PU detection can be formulated as a testing of two hypotheses: one is Hypothesis ${ℋ}_{0}$ when PU is absent, the other is hypothesis ${ℋ}_{1}$ when PU is present. The problem now is converted to distinguish between ${ℋ}_{0}$ and ${ℋ}_{1}$ in the following way:

(1) $$\begin{array}{l} {ℋ}_{0}\;:\;y=\Phi n\;\\ {ℋ}_{1}:y=\Phi \left(hs+n\;\right) \end{array}$$

where y is an M×1 compressive-sampled received signal. Φ is a compressive measurement matrix, i.e., $\Phi \in {\mathbb{R}}^{M\times N}\;,M\leq N$, which can be generated by Gaussian random matrices, Bernoulli random matrices, database-friendly matrices and random orthoprojection to ${\mathbb{R}}^{M}$. M and N are positive integer that depict size of the vector and matrix. s is an N×1 primary signal vector, and $s\in {\mathbb{R}}^{N}$ with power $\sigma_{s}^{2}={\left\|s\right\|}_{2}^{2}$, n is Additive White Gaussian Noise (AWGN) which is assumed as independent and identically distributed (i.i.d.) random process with zero mean and variance $\sigma_{n}^{2}$, i.e., $n∼N(0,\sigma_{n}^{2}I_{N})$, h is the channel gain between SU and PU. For our hypotheses, the joint probability distribution function (PDF) of compressed samples of received signal under hypothesis ${ℋ}_{0}$ and ${ℋ}_{1}$ can be derived as following:

(2) $$f_{0}(y|{ℋ}_{0})={\left(2\pi \right)}^{-N/2}{\left|\sigma_{n}^{2}\Phi \Phi^{T}\right|}^{-1/2}\exp\left(-\frac{1}{2}y^{T}{\left(\sigma_{n}^{2}\Phi \Phi^{T}\right)}^{-1}y\right)$$

(3) $$f_{1}(y|{ℋ}_{1})={\left(2\pi \right)}^{-N/2}{\left|\sigma_{n}^{2}\Phi \Phi^{T}\right|}^{-1/2}\exp\left(-\frac{1}{2}{\left(y-\Phi hs\right)}^{T}{\left(\sigma_{n}^{2}\Phi \Phi^{T}\right)}^{-1}\left(y-\Phi hs\right)\right)$$

According to the likelihood ratio test of Neyman–Pearson (NP) optimal decision rule [38,39]:

(4) $$\Lambda \left(y\right)=\frac{f_{1}(y|{ℋ}_{1})}{f_{0}(y|{ℋ}_{0})}\overset{{ℋ}_{1}}{\underset{{ℋ}_{0}}{≷}}\varphi$$

After taking logarithms on both sides, we can obtain an equivalent test that simplifies as:

(5) $$y^{T}{\left(\Phi \Phi^{T}\right)}^{-1}\Phi hs\overset{{ℋ}_{1}}{\underset{{ℋ}_{0}}{≷}}\sigma_{n}^{2}\log(\varphi )+\frac{1}{2}{\left(hs\right)}^{T}\Phi^{T}{\left(\Phi \Phi^{T}\right)}^{-1}\Phi hs:=\lambda$$

We now define the output of the compressive detector as:

(6) $$\upsilon :=y^{T}{\left(\Phi \Phi^{T}\right)}^{-1}\Phi hs$$

Since υ is a complete statistic for our detection problem, it contains complete information required to determine between ${ℋ}_{0}$ and ${ℋ}_{1}$. It is easy to prove that the PDF of *ν* can be approximated expressed by Gaussian distribution as follows:

(7) $${ℋ}_{0}:\upsilon ∼N\left(0,\sigma_{n}^{2}{\left(hs\right)}^{T}\Phi^{T}{\left(\Phi \Phi^{T}\right)}^{-1}\Phi hs\right)$$

(8) $${ℋ}_{1}:\upsilon ∼N\left({\left(hs\right)}^{T}\Phi^{T}{\left(\Phi \Phi^{T}\right)}^{-1}\Phi hs,\sigma_{n}^{2}{\left(hs\right)}^{T}\Phi^{T}{\left(\Phi \Phi^{T}\right)}^{-1}\Phi hs\right)$$

Thus, the false alarm probability $p_{f}$ and detection probability $p_{d}$ at SU can be computed by:

(9) $$p_{f}=\mathrm{Pr}\left(\upsilon >\lambda |{ℋ}_{0}\right)=Q\left(\frac{\lambda }{\sigma_{n}\sqrt{{\left(hs\right)}^{T}\Phi^{T}{\left(\Phi \Phi^{T}\right)}^{-1}\Phi hs}}\right)$$

(10) $$p_{d}=\mathrm{Pr}\left(\upsilon >\lambda |{ℋ}_{1}\right)=Q\left(\frac{\lambda -{\left(hs\right)}^{T}\Phi^{T}{\left(\Phi \Phi^{T}\right)}^{-1}\Phi hs}{\sigma_{n}\sqrt{{\left(hs\right)}^{T}\Phi^{T}{\left(\Phi \Phi^{T}\right)}^{-1}\Phi hs}}\right)$$

where the function $Q(x)=\frac{1}{\sqrt{2\pi }}\int_{x}^{+\infty }\exp(-y^{2}/2)dy$. From Equation (9), with target detection probability $p_{d}^{th}$ to provide sufficient protection to PU, $p_{f}$ is related $p_{d}^{th}$ to as follows:

(11) $$p_{f}=Q\left(Q^{-1}(p_{d}^{th})+\frac{\sqrt{{\left(hs\right)}^{T}\Phi^{T}{\left(\Phi \Phi^{T}\right)}^{-1}\Phi hs}}{\sigma_{n}}\right)$$

Firstly, considering a special case in which is an orthoprojector (i.e., $\Phi \Phi^{T}=I_{M}$), Theorem 1 gives the bound of the compressive detector performance.

**Theorem** **1.** *Let* Φ *be* Μ×Ν *random orthoprojector. Then, with probability at least of* 1−δ.

(12) $$Q\left(Q^{-1}(p_{d}^{th})+(1+\varepsilon )\sqrt{\mu \gamma }\right)\leq p_{f}\leq Q\left(Q^{-1}(p_{d}^{th})+(1-\varepsilon )\sqrt{\mu \gamma }\right)$$

*where* δ∈(0,1), $\varepsilon <\sqrt{12\log(2/\delta )/M}$, *the compressed sensing SNR* $\gamma =\frac{{\left\|hs\right\|}_{2}^{2}}{\sigma_{n}^{2}}$ *, and compression ratio* $\mu =\frac{M}{N}$.

**Proof**. Since Φ is an orthoprojector, Equation (11) can be rewritten:

(13) $$p_{f}=Q\left(Q^{-1}(p_{d}^{th})+\frac{{\left\|\Phi hs\right\|}_{2}}{\sigma_{n}}\right)$$

Based on the Johnson–Lindenstrauss (JL) lemma [31], then with probability at least 1−δ,

(14) $$(1-\varepsilon )\sqrt{\mu }{\left\|hs\right\|}_{2}\leq {\left\|\Phi hs\right\|}_{2}\leq (1+\varepsilon )\sqrt{\mu }{\left\|hs\right\|}_{2}$$

Combining Equations (13) and (14), the result follows. □

It can be concluded that Equation (12) tells us how much information we lose via compressed samples in terms of our ability to solve our detection problem, precisely. Moreover, it is absolutely feasible to use Equation (15) to predict the probability of false alarm because ε is so small that the impact on $p_{f}$ can be neglected with high probability. Although, for a given instance of Φ, the curve of relationship between $p_{f}$ and compression ratio M/N may be better or worse than the predicted curve by Equation (15).

(15) $$p_{f}\approx Q\left(Q^{-1}(p_{d}^{th})+\sqrt{\mu \gamma }\right)$$

**Figure 2:**
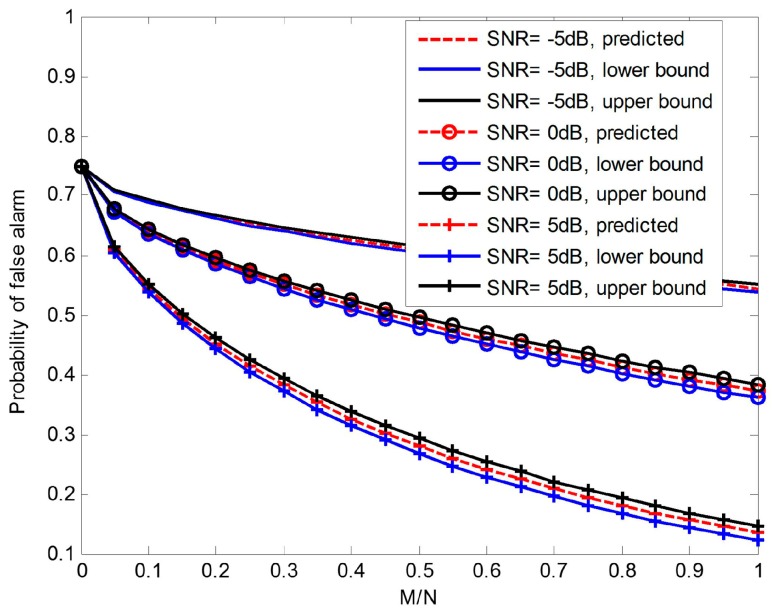
Effect of compression ratio on $p_{f}$ at several different SNR (signal to noise ratio) levels ($p_{d}^{th}=0.9$).

Obviously, it is tightly concentrated around the expected performance curve as shown in Figure 2.

Additionally, large numbers of trials of methods based on random orthoprojector, matrices with independent Gaussian entries and matrices with independent Bernoulli entries show that there is almost no difference among these three methods, and all perform highly consistent with expected. Actually, it is not necessary to limit Φ to be orthoprojector to satisfy the foregoing Theorem 1 and Equation (15).

During local compressed sensing, the *i-*th SU makes a binary decision (i.e., “1” for presence and “0” for absence) with probabilities of detection $p_{d,i}\;，\;i=1,2,...,K$ and false alarm $p_{f,i}\;，\;i=1,2,...,K$. Then, the FC combines all “one bit” decisions to make a global decision on the state of PU according to the hard counting fusion rule: if Λ<n, decide ${ℋ}_{0}$; else if Λ≥n, decide ${ℋ}_{1}$, where Λ is the number of SU which reports presence of PU, and  i=1,2,...,K is the decision threshold at FC. The expressions for cooperative false alarm probability $Q_{f}$ and cooperative detection probability $Q_{d}$ will be given as:

(16) $$Q_{f}=\sum_{j=n}^{K}\left(\begin{array}{l} K\\ \;j \end{array}\right)\;\times \left(\prod_{i=1}^{j}p_{f,i}\right)\times \left(\prod_{i=j+1}^{K}\left(1-p_{f,i}\right)\right)\;,\;Q_{d}=\sum_{j=n}^{K}\left(\begin{array}{l} K\\ \;j \end{array}\right)\;\times \left(\prod_{i=1}^{j}p_{d,i}\right)\times \left(\prod_{i=j+1}^{K}\left(1-p_{d,i}\right)\right)$$

In general, the counting fusion rule is broadly classified as OR fusion rule (OFR), AND fusion rule (AFR) and MAJORITY fusion rule (MFR).

For AFR, n takes K, we have:

(17) $$Q_{f\_AND}=\prod_{i=1}^{K}p_{f,i}\;,\;Q_{d\_AND}=\prod_{i=1}^{K}p_{d,i}$$

For OFR, n takes 1, we have:

(18) $$Q_{f\_OR}=1-\prod_{i=1}^{K}(1-p_{f,i})\;\;,\;Q_{d\_OR}=1-\prod_{i=1}^{K}\left(1-p_{d,i}\right)$$

For MFR, n takes the minimum integer greater than or equal to $\frac{K}{2}$, denoted by $\left\lfloor \frac{K}{2}\right\rfloor$. If $\Lambda <\left\lfloor \frac{K}{2}\right\rfloor$, decide ${ℋ}_{0}$; else if $\Lambda \geq \left\lfloor \frac{K}{2}\right\rfloor$, decide ${ℋ}_{1}$, and $\left\lfloor \frac{K}{2}\right\rfloor$ is the decision threshold at fusion center (FC). Then, we have:

(19) $$\begin{array}{l} Q_{f\_MFR}=\sum_{j=\left\lfloor \frac{K}{2}\right\rfloor }^{K}\left(\begin{array}{l} K\\ \;j \end{array}\right)\;\times \left(\prod_{i=1}^{j}p_{f,i}\right)\times \left(\prod_{i=j+1}^{K}\left(1-p_{f,i}\right)\right)\;\\ \;Q_{d\_MFR}=\sum_{j=\left\lfloor \frac{K}{2}\right\rfloor }^{K}\left(\begin{array}{l} K\\ \;j \end{array}\right)\times \left(\prod_{i=1}^{j}p_{d,i}\right)\times \left(\prod_{i=j+1}^{K}\left(1-p_{d,i}\right)\right) \end{array}$$

## 3. Novel Framework for Wideband Cooperative Spectrum Sensing

### 3.1. Analysis of SU Spectral Efficiency for Spectrum Sensing

**Figure 3:**
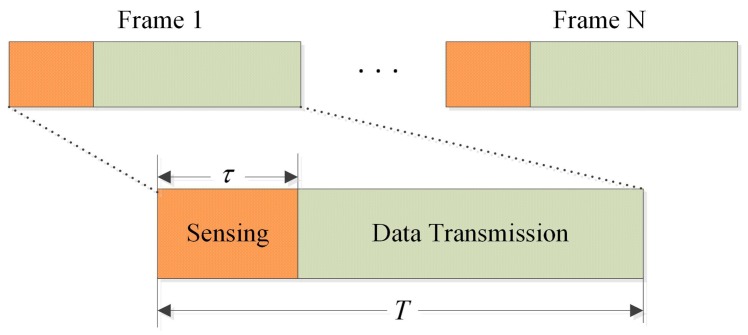
Frame structure of periodic spectrum sensing.

Due to the need for periodic spectrum sensing (PSS), a typical frame structure of a CR network will at least consist of a sensing slot and a data transmission slot, as depicted in Figure 3.

It is assumed that τ is used for implementing compressed spectrum sensing, and T−τ is reserved for transmission. In PSS, the *i-*th SU data transmission occurs in following two cases: correct detection absence of PU and missing detection presence of PU. When the former occurs, the amount of transmission data is $D_{i,1}=P_{{ℋ}_{0}}(1-p_{f,i})(T-\tau )r_{0}$, where $P_{{ℋ}_{0}}$ denotes the prior probability of the absence of PU and $r_{0}$ means transmission rate in the absence of PU, $r_{0}=Wlog_{2}\left(1+g^{2}\sigma_{s}^{2}/\sigma_{n}^{2}\right)$, W is the available channel bandwidth, $log_{2}$ means binary logarithm, and g is channel gain between SU. In the case of missing detection of PU, the amount of transmission data is $D_{i,2}=P_{{ℋ}_{1}}(1-p_{d,i})(T-\tau )r_{1}$, where $P_{{ℋ}_{1}}$ denotes the prior probability of the presence of PU and $r_{1}$ means transmission rate in the presence of PU, $r_{1}=W\;log_{2}\left(1+g^{2}\sigma_{s}^{2}/\sigma_{n}^{2}++h^{2}\sigma_{s}^{2}\right)$. Then, the transmission spectral efficiency at *i-*th SU is derived as:

(20) $$C=\frac{1}{WT}\left[P_{{ℋ}_{\;0}}(1-p_{f,i})(T-\tau )r_{0}+P_{{ℋ}_{1}}(1-p_{d,i})(T-\tau )r_{1}\right]$$

As to be highlighted, C is relied on the $p_{f,i}\;$ and $p_{d,i}$. It is equivalent to say that C is dependent on “*I*”, which is applicable for all any SU. Obviously, for a given frame length T, the longer the sensing time τ, the shorter the data transmission time T−τ. Since Q(x) is monotonically decreasing in x, for a target $p_{d,i}^{th}$ at SU, increasing the sensing time results in a lower false alarm probability. Hence, sensing time τ plays a crucial role in SU transmission spectral efficiency. Our fundamental purpose is to design the optimal sensing time τ to maximize the SU transmission spectral efficiency under the condition of sufficient protection to PU. Mathematically, the optimization problem can be described as:

(21) $$\begin{array}{l} \underset{\tau }{\max}:C\;\\ s.\;t.\;,\;p_{d,i}\geq p_{d,i}^{th} \end{array}$$

where $p_{d,i}^{th}$ is target detection probability with which PU is defined as being adequately protected.

**Theorem** **2.** C *is a decreasing function of* $p_{d}^{i}$.

**Proof**. For a given τ, we have:

(22) $$\frac{dC}{dp_{d,i}}=-\frac{1}{WT}\left(P_{{ℋ}_{0}}(T-\tau )r_{0}\frac{dp_{f,i}}{dp_{d,i}}+P_{{ℋ}_{1}}(T-\tau )r_{1}\right)$$

(23) $$\frac{dp_{f,i}}{dp_{d,i}}=\frac{dp_{f,i}}{d\lambda_{i}}/\frac{dp_{d,i}}{d\lambda_{i}}=\exp\left(-\frac{2\lambda_{i}-{\left\|\Phi hs\right\|}_{2}}{2\sigma_{n}^{2}}\right)>0$$

Obviously, $\frac{dC}{dp_{d,i}}<0$, C is a decreasing function of $p_{d,i}$. Thus, Theorem 2 is proven. When choosing the equality constraint $p_{d,i}=p_{d,i}^{th}$, the optimal spectral efficiency can be achieved with compression ratio $\mu_{i}$ and sensing SNR $\gamma_{i}$ at the *i-*th SU. Then, we have:

(24) $$C=\frac{(T-\tau )r_{0}}{WT}P_{{ℋ}_{0}}\left\{1-Q\left(Q^{-1}(p_{d,i}^{th})+\sqrt{\mu_{i}\gamma_{i}}\right)\right\}+\frac{(T-\tau )r_{1}}{WT}P_{{ℋ}_{1}}(1-p_{d,i}^{th})□\;$$

**Theorem** **3.** *For any target detection probability* $p_{d,i}^{th}$, *there exists one optimal sensing time* $\tau_{opt}$ *that yields the maximal SU spectral efficiency*.

**Proof**. For a given $p_{d,i}^{th}$, 0<τ<T and $\mu_{i}=\frac{f_{s}\times \Delta t_{i}}{N}=\frac{f_{s}\times \tau }{N}$, in which $f_{s}$ denotes sampling frequency in compressed domain, $\Delta t_{i}$ is compressed sensing time, then we have:

(25) $$\begin{array}{c} \nabla C(\tau )=-\frac{r_{0}}{WT}P_{{ℋ}_{0}}\left\{1-Q\left(Q^{-1}(p_{d,i}^{th})+\sqrt{\frac{f_{s}\gamma_{i}}{N}\tau }\right)\right\}-\frac{r_{1}}{WT}P_{{ℋ}_{1}}(1-p_{d,i}^{th})\\ +\frac{(T-\tau )r_{0}P_{{ℋ}_{0}}}{2WT}\sqrt{\frac{f_{s}\gamma_{i}}{2\pi N\tau }}\exp\left\{-\frac{1}{2}{\left(Q^{-1}(p_{d,i}^{th})+\sqrt{\frac{f_{s}\gamma_{i}}{N}\tau }\right)}^{2}\right\} \end{array}$$

Obviously,

(26) $$\underset{\tau \to 0}{\lim}\nabla C(\tau )=+\infty$$

(27) $$\underset{\tau \to T}{\lim}\nabla C(\tau )=-\frac{r_{0}}{WT}P_{{ℋ}_{0}}\left\{1-Q\left(Q^{-1}(p_{d,i}^{th})+\sqrt{\frac{f_{s}\gamma_{i}}{N}\tau }\right)\right\}-\frac{r_{1}}{WT}P_{{ℋ}_{1}}(1-p_{d,i}^{th})$$

Because Q(x) is the monotonously decreasing function in x, we have:

(28) $$Q\left(Q^{-1}(p_{d,i}^{th})+\sqrt{\frac{f_{s}\gamma_{i}}{N}\tau }\right)<Q\left(Q^{-1}(p_{d,i}^{th})\right)=p_{d,i}^{th}$$

Thus,

(29) $$\underset{\tau \to T}{\lim}\nabla C(\tau )<-\frac{r_{0}}{WT}P_{{ℋ}_{0}}\left(1-p_{d,i}^{th}\right)-\frac{r_{1}}{WT}P_{{ℋ}_{1}}(1-p_{d,i}^{th})<0\;□$$

Equations (26) and (29) indicate that C increases when τ is small and decreases when τ approaches T. Hence, there exists one extremum point of C for τ∈(0, T) which satisfies the constraint on ∇C(τ)=0. Furthermore, we have:

(30) $$\begin{array}{l} \nabla^{2}C(\tau )=-[\frac{r_{0}P_{{ℋ}_{0}}}{2WT}\sqrt{\frac{f_{s}\gamma_{i}}{2\pi N\tau }}+\frac{r_{0}P_{{ℋ}_{0}}}{4\sqrt{2\pi }WT}\frac{f_{s}\gamma_{i}}{N}\frac{\left(T-\tau \right)}{\tau }\\ \times \left(Q^{-1}(p_{d,i}^{th})+\sqrt{\frac{f_{s}\gamma_{i}}{N}\tau }\right)+\frac{r_{0}P_{{ℋ}_{0}}}{4WT}\sqrt{\frac{f_{s}\gamma_{i}}{2\pi N}}\frac{T+\tau }{\tau \sqrt{\tau }}]\\ \exp\left\{-\frac{1}{2}{\left(Q^{-1}(p_{d,i}^{th})+\sqrt{\frac{f_{s}\gamma_{i}}{N}\tau }\right)}^{2}\right\} \end{array}$$

Eventually, we have $\nabla^{\;2}C(\tau )<0$ for τ∈(0, T), which means that C is a convex function of τ. Hence, there exists one optimal sensing time $\tau_{opt}\in (0,\;T)$ that maximizes the SU spectral efficiency. Thus, Theorem 3 is proven.

### 3.2. Novel Wideband Cooperative Spectrum Sensing Framework

**Figure 4:**
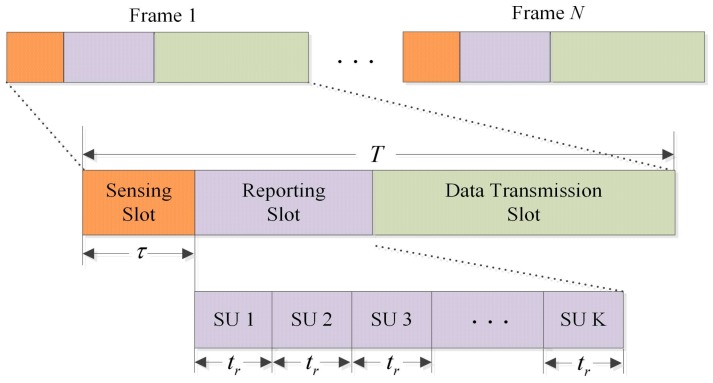
General TDMA-based cooperative spectrum sensing framework.

In order to avoid interfering to PU, it necessitates an efficient and reliable detection method of PU. The scheme of CSS has been proposed to mitigate the impact of fading and shadowing. The secondary system consists of K SUs and one FC. Figure 4 illustrates the general TDMA-based CSS frame structure designed for a CR network with PSS, where each frame consists of a compressed sensing (CS) slot, a reporting slot and a data transmission slot, ignoring the time consumed by FC to process the collected data and to broadcast the final decision. Supposing the frame duration is T, the sensing duration is τ and the individual reporting duration is $t_{r}$. Prior to data transmission, CSS is performed synchronously by SU at the beginning of the frame, then each SU reports local sensing results to FC in a single reporting time slot following sensing slot to avoid transmission conflict. Finally, the FC makes a global decision on the state of PU. Only when the absence of PU is detected can transmission slot be used. Otherwise, SU must vacate the frequency band immediately and perform spectrum sensing again.

Cooperation among SU can greatly improve sensing performance, however, it also introduces additional overhead since the sensing results need to be reported to FC periodically. In the conventional frame structure, the SU can sense the PU before sensing results reporting and data transmission. A serious problem is that when the incumbent SU reports its local sensing results, other SU have no any manipulation until allocated reporting slot arrival. There is a waste of $K(K-1)t_{r}$ reporting duration that does not make any contribution to the performance of spectrum sensing. Therefore, the general cooperative sensing frame structure is inefficient. In this section, we propose a high-efficiency CSS framework with optimal scheduling strategy to improve the performance of cooperative sensing without introducing additional time overhead. The core idea of the novel framework is that when the incumbent SU is reporting the sensing results, its succeeding SU continue local compressed sensing until their turns to report, which aims at extending the sensing duration as long as possible by fully utilizing the reporting slot.

Considering the effects of dynamic and time-varying spectrum environment on CSS performance, most recent works [14,15,16,17,27,29,30,31,32] regard PU as either present or absent for the whole sensing frame length. This is a reasonable model when the PU has low traffic. Furthermore, in respect of high traffic, Shi et al. [34] and Tang et al. [40] analyzed the PU’s random arrival or departure during the sensing period. Although Shi et al. [34] considered the case that PU could become busy again during SU’s transmission, the sensing period is assumed to be short as compared to the average busy and idle periods of PU from the aspect of traffic with stringent quality of service (QoS) requirements. For simplicity, Tang et al. [40] considered a common case that the status of licensed user (LU) is not changing very frequent and ignores the asynchronous activities of LU and smart devices (SD). Additionally, the effects of the PU’s random departure/arrival behaviors on spectrum sensing are minimal [41]. On this basis, it is assumed that the PU does not leave or arrive within *N* frames for CSS frame structure with individual frame length of 160 ms [35], where the value of *N* is determined by the application scenarios. Thus, our proposed novel CSS frame structure can be applied to the scenario where the PU does not leave or arrive within one frame, which is the precondition of the proposed solution.

**Figure 5:**
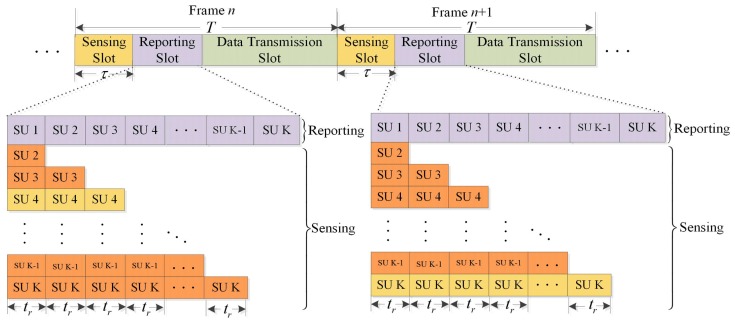
Novel cooperative spectrum sensing frame structure.

As depicted in Figure 5, the *i-*th SU reporting time can be utilized for its following SU sensing. In other words, for the *i-*th SU, the $(i-1)t_{r}$ calculated in reporting slot can be employed for local compressed sensing. Hence, the equivalent sensing time of the *i-*th SU is $\tau +(i-1)t_{r}$. For example, the total duration $\tau +3\;t_{r}$ and $\tau +(K-1)t_{r}$ in the yellow region of Figure 5 denotes the equivalent sensing time of SU 4 and SU K, respectively.

In summation, the equivalent compressed sensing time for the *i*-th SU is denoted as:

(31) $$\Delta t_{i}=\tau +(i-1)t_{r}\;,\;i=1,2,...,K$$

The quantity of compressed samples at the *i*-th SU is formulated as:

(32) $$M_{i}=\Delta \;t_{i}f_{s}=(\tau +(i-1)t_{r})f_{s}\;,\;i=1,2,...,K$$

Therefore, the equivalent compression ratio at the *i*-th SU is

(33) $$\mu_{i}=\frac{M_{i}}{N}=\frac{\Delta t_{i}f_{s}}{N}=\frac{(\tau +(i-1)t_{r})f_{s}}{N}\;,\;i=1,2,...,K$$

As depicted in Figure 5, the sensing time for *j*-th SU is $\tau +(j-1)t_{r}$, which does not exceeds $\tau +Kt_{r}$. Thus, the sensing time for any SU is restricted in the open interzone of $\left(0,\tau +Kt_{r}\right)$. The data transmission occurs in the closed interzone of $[\tau +Kt_{r},T]$. There is a good reason to believe that *i*-th SU transmission and the *j*-th SU sensing cannot happen at the same time. Thus, the signal that the *j*-th SU senses does not include the signal transmitted from *i*-th SU. Another situation to be highlighted is coincidence of the *i*-th SU reporting and the *j*-th SU sensing. According to the description in Figure 1, the *i*-th SU reports a binary decision (i.e., “1” for presence and “0” for absence) in the reporting link, whereas the *j*-th SU senses in the sensing link. In this case, they are using different frequency channel. It is true that sensing link and reporting link are independent physical channels.

It can be seen clearly that each SU except SU 1 gets much longer compressed sensing time and higher compression ratio without additional time overhead compared with the general frame structure. Thus, SU spectral efficiency can be increased due to the reduced false alarm probability. In summation, it is the improvement of overall sensing performance that contributes to spectral efficiency, as demonstrated in aforementioned argument.

## 4. Optimal Sensing Settings for SU Spectral Efficiency

### 4.1. Reporting Scheduling Strategy

In the proposed CSS framework, if sensing SNR $\gamma_{i}$ received at each SU is identical, the reporting order of cooperative SU makes no difference to sensing performance. However, the primary signal received at different SU are considered to be unequal due to varying channel conditions and diverse path loss caused by independent distance from PU to SU. To achieve the optimal cooperative sensing performance, we propose reporting scheduling strategy based on the received $\gamma_{i}$ at the *i*-th SU.

In general, the optimal reporting scheduling strategy changes with the varied fusion rules. In this section, the optimal scheduling strategy for AFR, OFR and MFR are investigated. The sensing false alarm probability and detection probability dictate the feasibility of SU operation, and there is typically a tradeoff between the two. The PU would prefer a high detection probability to alleviate interference. However, the SU would prefer a low false alarm probability to use the PU’s frequency band for secondary data transmission, thereby increasing wireless efficiency. Generally, the Neyman–Pearson (NP) detector is the decision rule that maximizes detection probability $P_{D}$ subject to the constraint that false alarm probability $P_{F}\leq \alpha$. In practice, it is of interest to optimize performance by minimizing the false alarm probability given a target detection probability. Our object is to achieve the maximal spectral efficiency with sufficient protection to PU, which is equivalent to minimize the false alarm probability with other parameters constant. In respect of different fusion rules, our main task is to minimize the probability of cooperative false alarm $Q_{f}$ with the target cooperative detection probability $Q_{d}^{th}$ during the following formulas deduction.

When AFR is applied at FC, according to Equation (17), the individual target detection probability at the *i*th SU is $p_{d,i}^{th}=\sqrt[{K}]{Q_{d}^{th}}$, then the individual false alarm probability can be derived from Equation (15):

(34) $$p_{f,i}=Q\left(Q^{-1}(p_{d,i}^{th})+\sqrt{\frac{1}{N}M_{i}\gamma_{i}}\right)$$

Mathematically, the objective optimization function can be modeled as:

(35) $$\begin{array}{ll} min\;: & Q_{f\_AND}\;\\ s.\;t.\;: & Q_{f\_AND}\;=\prod_{i=1}^{K}p_{f,\;i}\\ & \;Q_{d\_AND}\geq Q_{d}^{th} \end{array}$$

Primarily, we take K=2 as an entrance to analyze the optimal reporting scheduling strategy. It is equivalent to sort SNR {γ, γ+ Δγ} for the given compressed samples {M, M+ΔM} with $A_{2}^{2}$ kinds of permutation methods: (γ,M;γ+Δγ,M+ΔM) and (γ,M+ΔM;γ+Δγ,M). The false alarm probabilities of two cooperative SUs with AFR are computed by:

(36) $$Q_{f\_AND}^{\left(2\right)}(\gamma ,M;\gamma +\Delta \gamma ,M+\Delta M)=p_{f,1}(\gamma ,M)p_{f,2}(\gamma +\Delta \gamma ,M+\Delta M)$$

(37) $$Q_{f\_AND}^{\left(2\right)}(\gamma ,M+\Delta M;\gamma +\Delta \gamma ,M)=p_{f,1}(\gamma ,M+\Delta M)p_{f,2}(\gamma +\Delta \gamma ,M)$$

It can be seen from Equation (15) that individual $p_{f,i}$ decreases as the number of compressed samples $M_{i}$ or SNR $\gamma_{i}$ increases with the given $p_{d,i}^{th}$. Hence, we have $\frac{\partial p_{f,i}(\gamma_{i},M_{i})}{\partial \gamma_{i}}<0$, $\frac{\partial p_{f,i}(\gamma_{i},M_{i})}{\partial M_{i}}<0$ and $p_{f,1}(\gamma ,M)=p_{f,2}(\gamma ,M)$. According to the binary function differential equation, $Q_{f\_AND}^{\left(2\right)}(\gamma ,M;\gamma +\Delta \gamma ,M+\Delta M)$ and $Q_{f\_AND}^{\left(2\right)}(\gamma ,M+\Delta M;\gamma +\Delta \gamma ,M)$ can be approximately expressed as:

(38) $$\begin{array}{l} Q_{f\_AND}^{\left(2\right)}(\gamma ,M;\gamma +\Delta \gamma ,M+\Delta M)=p_{f,1}(\gamma ,M)p_{f,2}(\gamma +\Delta \gamma ,M+\Delta M)\\ =p_{f,1}(\gamma ,M)p_{f,2}(\gamma ,M)+p_{f,1}(\gamma ,M)\frac{\partial p_{f,2}(\gamma ,M)}{\partial \gamma }\Delta \gamma +p_{f,1}(\gamma ,M)\frac{\partial p_{f,2}(\gamma ,M)}{\partial M}\Delta M \end{array}$$

(39) $$\begin{array}{l} Q_{f\_\mathrm{AND}}^{\left(2\right)}(\gamma ,M+\Delta M;\gamma +\Delta \gamma ,M)=p_{f,1}(\gamma ,M+\Delta M)p_{f,2}(\gamma +\Delta \gamma ,M)\\ =p_{f,1}(\gamma ,M)p_{f,2}(\gamma ,M)+p_{f,1}(\gamma ,M)\frac{\partial p_{f,2}(\gamma ,M)}{\partial \gamma }\Delta \gamma +p_{f,2}(\gamma ,M)\frac{\partial p_{f,1}(\gamma ,M)}{\partial M}\Delta M\\ \;+\frac{\partial p_{f,1}(\gamma ,M)}{\partial M}\frac{\partial p_{f,2}(\gamma ,M)}{\partial \gamma }\Delta \gamma \Delta M \end{array}$$

Then, we have

(40) $$Q_{f\_\mathrm{AND}}^{\left(2\right)}(\gamma ,M;\gamma +\Delta \gamma ,M+\Delta M)\leq Q_{f\_AND}^{\left(2\right)}(\gamma ,M+\Delta M;\gamma +\Delta \gamma ,M)$$

In the case of two SUs, when the sensing results of SU with higher received SNR is reported later, which gets more compressed samples under AFR, it will achieve better cooperative false alarm probability. The reporting turn of K SUs can be attained by comparing that of any two SUs. Thus, in order to achieve better performance of spectrum reuse with the sufficient protection to PU, reporting sensing results to FC in the ascending sort of received SNR $\gamma_{i}$ would be the obligatory scheduling strategy for the proposed novel CSS framework.

When OFR is used at FC, according to Equation (18), the individual target detection probability at the *i-*th SU is $p_{d,i}^{th}=1-\sqrt[{K}]{1-Q_{d}^{th}}$ then the individual false alarm probability $p_{f,i}$ can be derived from Equation (15). Mathematically, the optimization goal can be formulized as:

(41) $$\begin{array}{ll} min\;: & Q_{f\_OR}\;\\ s.t.\;: & Q_{f\_OR}=1-\prod_{i=1}^{K}\left(1-p_{f,i}\right)\\ & \;Q_{d\_OR}\geq Q_{d}^{th} \end{array}$$

Similarly, we take two SUs to investigate the reporting scheduling strategy for the novel CSS framework. The cooperative false alarm probabilities of two SUs under OFR are, respectively, given as:

(42) $$Q_{f\_\mathrm{OR}}^{\left(2\right)}(\gamma ,M;\gamma +\Delta \gamma ,M+\Delta M)=1-\left(1-p_{f,1}(\gamma ,M)\right)\left(1-p_{f,2}(\gamma +\Delta \gamma ,M+\Delta M)\right)$$

(43) $$Q_{f\_\mathrm{OR}}^{\left(2\right)}(\gamma ,M+\Delta M;\gamma +\Delta \gamma ,M)=1-\left(1-p_{f,1}(\gamma ,M+\Delta M)\right)\left(1-p_{f,2}(\gamma +\Delta \gamma ,M)\right)$$

Theoretically, $Q_{f\_\mathrm{OR}}^{\left(2\right)}(\gamma ,M;\gamma +\Delta \gamma ,M+\Delta M)$ and $Q_{f\_\mathrm{OR}}^{\left(2\right)}(\gamma ,M+\Delta M;\gamma +\Delta \gamma ,M)$ can be approximately represented as:

(44) $$\begin{array}{l} Q_{f\_\mathrm{OR}}^{\left(2\right)}(\gamma ,M;\gamma +\Delta \gamma ,M+\Delta M)=1-\left(1-p_{f,1}(\gamma ,M)\right)\left(1-p_{f,2}(\gamma +\Delta \gamma ,M+\Delta M)\right)\\ =p_{f,1}(\gamma ,M)+p_{f,2}(\gamma ,M)-p_{f,1}(\gamma ,M)p_{f,2}(\gamma ,M)\\ \;+\left(1-p_{f,1}(\gamma ,M)\right)\left(\frac{\partial p_{f,2}(\gamma ,M)}{\partial M}\Delta M+\frac{\partial p_{f,2}(\gamma ,M)}{\partial \gamma }\Delta \gamma \right) \end{array}$$

(45) $$\begin{array}{l} Q_{f\_\mathrm{OR}}^{\left(2\right)}(\gamma ,M+\Delta M;\gamma +\Delta \gamma ,M)=1-\left(1-p_{f,1}(\gamma ,M+\Delta M)\right)\left(1-p_{f,2}(\gamma +\Delta \gamma ,M)\right)\\ =p_{f,1}(\gamma ,M)+p_{f,2}(\gamma ,M)-p_{f,1}(\gamma ,M)p_{f,2}(\gamma ,M)+\left(1-p_{f,1}(\gamma ,M)\right)\\ \;\times \left(\frac{\partial p_{f,2}(\gamma ,M)}{\partial M}\Delta N+\frac{\partial p_{f,2}(\gamma ,M)}{\partial \gamma }\Delta \gamma \right)-\frac{\partial p_{f,2}(\gamma ,M)}{\partial M}\frac{\partial p_{f,2}(\gamma ,M)}{\partial \gamma }\Delta \gamma \Delta M \end{array}$$

Apparently, we have

(46) $$Q_{f\_\mathrm{OR}}^{\left(2\right)}(\gamma ,M+\Delta M;\gamma +\Delta \gamma ,M)\leq Q_{f\_\mathrm{OR}}^{\left(2\right)}(\gamma ,M;\gamma +\Delta \gamma ,M+\Delta M)$$

In the case of two SUs under OFR, it performs better in cooperative false alarm probability when higher SNR gets less compressed samples. Then we can attain K SUs reporting turn by comparing any two SUs. In order to utilize spectrum fully with the sufficient protection to PU, it is required to deliver sensing results to FC in the descending sort of received SNR $\gamma_{i}$ for the novel CSS framework.

When MFR is applied at the FC, the analysis process of optimal reporting scheduling strategy is similar to AFR and OFR above mentioned. For MFR, we consider the majority rule as n takes the minimum integer greater than or equal to $\frac{N}{2}$, denoted by $\left\lfloor \frac{N}{2}\right\rfloor$, so n takes 1 when N=2, the MFR will simplify to OFR with two SUs, thus, the scheduling strategy is dependent on any of two SUs’ reporting turn based on sensing SNR. It is also desired to report sensing results to FC in the descending sort of received SNR $\gamma_{i}$ to achieve better performance of spectrum utilization with sufficient protection to PU.

### 4.2. Optimal Sensing Settings of the Novel ST-CSS Framework with Maximal SU Spectral Efficiency

As analyzed in the novel CSS scheme, the *i-*th SU equivalent compressed sensing time amounts to $\tau +(i-1)\;t_{r}$. To overcome the adverse effects on detection performance in time-varying channels, time diversity gain can be achieved through combining multi-slot sensing results. It is assumed that individual sensing time is divided into L slots, and interval of each slot is $\Delta t_{i}=\left(\tau +(i-1)t_{r}\right)/L$ The compressed samples at the *l-*th slot of *i-*th SU is given as: $y_{i,l}=\eta \Phi_{i,l}h_{i,l}s_{i,l}+\Phi_{i,l}n_{i,l}$, where η=0,1 denotes the absence and presence of PU, $\Phi_{\;i,l}$ is the measurement matrix at *l*th slot of *i-*th SU, $s_{i,l}$ is the signal received at *l-*th slot of *i-*th SU from PU and the signal power is constant over the L slots, i.e., $\sigma_{s,i}^{2}={\left\|s_{i,l}\right\|}_{2}^{2}$, the same as noise power $\sigma_{n}^{2}={\left\|n_{i,l}\right\|}_{2}^{2}$, $h_{i,l}$ is the channel gain between *l*th slot of *i-*th SU and PU. The output of compressive detector is given as: $\vartheta_{i,l}\;=y_{i,l}^{T}\Phi_{i,l}h_{i,l}s_{i,l}$. The aggregate compressed statistics $\upsilon {\;}_{i}$ used for local decision of *i-*th SU is obtained by summing $\vartheta_{i,l}$ with its corresponding weight $\omega_{i,l}$ as follows:

(47) $$\upsilon_{\;i}=\sum_{l=1}^{L}\omega_{i,l}\vartheta_{i,l}$$

Note that $\sum_{l=1}^{L}\omega_{i,l}^{2}=1$. Then, the mean and variance of $\upsilon_{\;i}$ at η=0 and η=1 are derived as:

(48) $$\left\{ \begin{array}{l} E\left(\upsilon_{i}|\eta =0\right)=0\\ Var\left(\upsilon_{\;i}|\eta =0\right)=\frac{\left(\tau +(i-1)t_{r}\right)f_{s}}{NL}\sigma_{n}^{2}\sigma_{s,i}^{2}\sum_{l=1}^{L}\omega_{i,l}^{2}{\left|h_{i,l}\right|}^{2}\\ E\left(\upsilon_{i}|\eta =1\right)=\frac{\left(\tau +(i-1)t_{r}\right)f_{s}}{NL}\sigma_{s,i}^{2}\sum_{l=1}^{L}\omega_{i,l}{\left|h_{i,l}\right|}^{2}\\ Var\left(\upsilon_{\;i}|\eta =1\right)=\frac{\left(\tau +(i-1)t_{r}\right)f_{s}}{NL}\sigma_{n}^{2}\sigma_{s,i}^{2}\sum_{l=1}^{L}\omega_{i,l}^{2}{\left|h_{i,l}\right|}^{2} \end{array}\right.$$

From now on, we call the above proposed scheme spatiotemporal CSS (ST-CSS), which combines the space diversity gain with time diversity gain. Thus, the false alarm and detection probabilities of the weighted ST-CSS at *i-*th SU is given as:

(49) $${\hat{p}}_{f,i}=Q\left(\frac{\lambda_{i}-E\left(\upsilon_{i}|\eta =0\right)}{\sqrt{Var\left(\upsilon_{i}|\eta =0\right)}}\right)=Q\left(\frac{\lambda_{i}}{\sqrt{\frac{\left(\tau +(i-1)t_{r}\right)f_{s}}{NL}\sigma_{n}^{2}\sigma_{s,i}^{2}\sum_{l=1}^{L}\omega_{i,l}^{2}{\left|h_{i,l}\right|}^{2}}}\right)$$

(50) $${\hat{p}}_{d,i}=Q\left(\frac{\lambda_{i}-E\left(\upsilon_{i}|\eta =1\right)}{\sqrt{Var\left(\upsilon_{i}|\eta =1\right)}}\right)=Q\left(\frac{\lambda_{i}-\frac{\left(\tau +(i-1)t_{r}\right)f_{s}}{NL}\sigma_{s,i}^{2}\sum_{l=1}^{L}\omega_{i,l}{\left|h_{i,l}\right|}^{2}}{\sqrt{\frac{\left(\tau +(i-1)t_{r}\right)f_{s}}{NL}\sigma_{n}^{2}\sigma_{s,i}^{2}\sum_{l=1}^{L}\omega_{i,l}^{2}{\left|h_{i,l}\right|}^{2}}}\right)$$

Combining Equation (49) with Equation (50), ${\hat{p}}_{f,i}$ is related to ${\hat{p}}_{d,i}$ as follows:

(51) $${\hat{p}}_{f,i}=Q\left(Q^{-1}({\hat{p}}_{d,i})+\frac{\sum_{l=1}^{L}\omega_{i,l}{\left|h_{i,l}\right|}^{2}}{\sqrt{\sum_{l=1}^{L}\omega_{i,l}^{2}{\left|h_{i,l}\right|}^{2}}}\sqrt{\frac{\left(\tau +(i-1)t_{r}\right)f_{s}}{NL}\times \frac{\sigma_{s,i}^{2}}{\sigma_{n}^{2}}}\right)$$

In order to achieve the maximal spectrum utilization efficiency for the proposed novel ST-CSS scheme, we seek to minimize ${\hat{p}}_{f,i}$, the optimization problem is formulated as follows:

(52) $$\underset{\omega_{i,1},\omega_{i,2},...,\omega_{i,L}}{\min}{\hat{p}}_{f,i}=Q\left(Q^{-1}({\hat{p}}_{d,i})+\frac{\sum_{l=1}^{L}\omega_{i,l}{\left|h_{i,l}\right|}^{2}}{\sqrt{\sum_{l=1}^{L}\omega_{i,l}^{2}{\left|h_{i,l}\right|}^{2}}}\sqrt{\frac{\left(\tau +(i-1)t_{r}\right)f_{s}}{NL}\times \frac{\sigma_{s,i}^{2}}{\sigma_{n}^{2}}}\right)$$

For a given ${\hat{p}}_{d,i}$, because Q (⋅) is a monotonously decreasing function, Equation (52), is converted to maximize

(53) $$\underset{\omega_{i,1},\omega_{i,2},...,\omega_{i,L}}{\max}\;\frac{\sum_{l=1}^{L}\omega_{i,l}{\left|h_{i,l}\right|}^{2}}{\sqrt{\sum_{l=1}^{L}\omega_{i,l}^{2}{\left|h_{i,l}\right|}^{2}}}$$

In order to minimize ${\hat{p}}_{f,i}$ the optimal weighted coefficients should be obtained by letting:

(54) $$\omega_{i,l}=\frac{{\left|h_{i,l}\right|}^{2}}{\sqrt{\sum_{l=1}^{L}\;{\left|h_{i,l}\right|}^{4}}}\;1\leq l\leq L$$

Then, with a given ${\hat{p}}_{d,i}$ at *i-*th SU, combining Equations (52) and (54), the minimum probability of false alarm ${\hat{p}}_{f,i}$ is deduced as following:

(55) $${\hat{p}}_{f,i}=Q\left(Q^{-1}({\hat{p}}_{d,i})+\frac{\sum_{l=1}^{L}{\left|h_{i,l}\right|}^{4}}{\sqrt{\sum_{l=1}^{L}{\left|h_{i,l}\right|}^{6}}}\sqrt{\frac{\left(\tau +(i-1)t_{r}\right)f_{s}}{NL}\times \frac{\sigma_{s,i}^{2}}{\sigma_{n}^{2}}}\right)$$

With counting hard fusion rule deployed at FC, the final probabilities of detection and false alarm of the proposed ST-CSS scheme are given by:

(56) $${\hat{Q}}_{f}=\sum_{j=n}^{K}\left(\begin{array}{l} K\\ \;j \end{array}\right)\;{\hat{p}}_{f,i}^{j}{\left(1-{\hat{p}}_{f,i}\right)}^{K-j}$$

(57) $${\hat{Q}}_{d}=\sum_{j=n}^{K}\left(\begin{array}{l} K\\ \;j \end{array}\right)\;{\hat{p}}_{d,i}^{j}{\left(1-{\hat{p}}_{d,i}\right)}^{K-j}$$

Thus, for the proposed novel ST-CSS scheme with hard decision fusion rule, similar to Equation (20), and the latency $t_{0}$ due to the very long distances that the signals must travel into space and back, which comprises of transmission from SU to FC and broadcasting the final decision on the state of PU from FC to SU is considered. The average SU transmission spectral efficiency is:

(58) $$\hat{C}=\frac{T-\tau -Kt_{r}-t_{0}}{WT}\left(P_{{ℋ}_{0}}(1-{\hat{Q}}_{f})r_{0}+P_{{ℋ}_{1}}(1-{\hat{Q}}_{d})r_{1}\right)$$

In the scenario of mobile satellite communication confronted with the problem of frequency scarcity, the degraded spectrum utilization will result in a greatly decreasing communication capacity or even interruption for mobile satellite service. In the proposed novel ST-CSS scheme, the interference to PU caused by the secondary network mainly results from missed detection of PU, a high probability of detection is required to ensure tolerable interference caused by SUs. For example, in IEEE 802.22 WRAN, the target detection probability is selected to be 90% for an SNR of −20 dB for the primary signal at SU [28]. We focus on the joint optimization of the reporting scheduling strategy and fusion scheme to maximize the transmission spectral efficiency with adequate protection to PU. The optimization problem can be modeled as:

(59) $$\begin{array}{l} \max:\hat{C}\\ s.\;t.\;,\;{\hat{Q}}_{d}\geq {\hat{Q}}_{d}^{th}\;;\;1\leq n\leq K \end{array}$$

**Theorem** **4.** $\hat{C}$ *is a decreasing function of* ${\hat{Q}}_{d}$.

**Proof**. For the given n, we have:

(60) $$\frac{d\hat{C}}{d{\hat{Q}}_{d}}=-\frac{T-\tau -Kt_{r}-t_{0}}{WT}\left(P_{{ℋ}_{0}}r_{0}\frac{d{\hat{Q}}_{f}}{d{\hat{Q}}_{d}}+P_{{ℋ}_{1}}r_{1}\right)$$

(61) $$\begin{array}{cc} \frac{d{\hat{Q}}_{f}}{d{\hat{Q}}_{d}} & =\frac{d{\hat{Q}}_{f}}{d{\hat{p}}_{f,i}}/\frac{d{\hat{Q}}_{d}}{d{\hat{p}}_{d,i}}\times \frac{d{\hat{p}}_{f,i}}{d{\hat{p}}_{d,i}}\\ & =\frac{K\left(\begin{array}{l} K-1\\ \;n-1 \end{array}\right){\left({\hat{p}}_{f,i}\right)}^{n-1}{\left(1-{\hat{p}}_{f,i}\right)}^{K-n}}{K\left(\begin{array}{l} K-1\\ \;n-1 \end{array}\right){\left({\hat{p}}_{d,i}\right)}^{n-1}{\left(1-{\hat{p}}_{d,i}\right)}^{K-n}}\times \frac{d{\hat{p}}_{f,i}}{d{\hat{p}}_{d,i}}\\ & ={\left(\frac{{\hat{p}}_{f,i}}{{\hat{p}}_{d,i}}\right)}^{n-1}{\left(\frac{1-{\hat{p}}_{f,i}}{1-{\hat{p}}_{d,i}}\right)}^{K-n}\times \frac{d{\hat{p}}_{f,i}}{d{\hat{p}}_{d,i}} \end{array}$$

According to Equations (49) and (50), we have:

(62) $$\frac{d{\hat{p}}_{f,i}}{d{\hat{p}}_{d,i}}=\frac{d{\hat{p}}_{f,i}}{d\lambda_{i}}/\frac{d{\hat{p}}_{d,i}}{d\lambda_{i}}>0$$

Ultimately, we have $\frac{d{\hat{Q}}_{f}}{d{\hat{Q}}_{d}}>0$ and $\frac{d\hat{C}}{d{\hat{Q}}_{d}}<0$. Then, $\hat{C}$ is the decreasing function of ${\hat{Q}}_{d}$. Theorem 4 is proven. □

The maximal spectral efficiency of the proposed novel ST-CSS scheme can be achieved if and only if when the following relationship establishes:

(63) $$\Gamma \left({\hat{p}}_{d,i}\right)={\hat{Q}}_{d}-{\hat{Q}}_{d}^{th}=\sum_{j=n}^{K}\left(\begin{array}{l} K\\ \;j \end{array}\right){\hat{p}}_{d,i}^{j}{\left(1-{\hat{p}}_{d,i}\right)}^{K-j}-{\hat{Q}}_{d}^{th}=0$$

In this section, we propose an algorithm based on Equations (55)–(63) to find the maximal spectral efficiency of the proposed novel ST-CSS scheme with its corresponding optimal reporting scheduling strategy when n takes 1, ⌊K/2⌋, and K, respectively. Replacing ${\hat{p}}_{d,i}$ in Equation (55) with the root of Equation (63), we can get the minimal cooperative false alarm from Equation (56), as well the optimal spectral efficiency from Equation (58). In the case of n=1 and n=K, the root of Equation (63) can be calculated via exponential operation. The key of the problem lies in how to calculate the root of Equation (63) when n=⌊K/2⌋. Several algorithms, such as Newton-Raphson, SECOND and Bi-Section, have been employed to seek the root numerically. The Newton-Raphson has a faster convergence rate than the other algorithms since the explicit expression of derivative of Γ over ${\hat{p}}_{d,i}$ exists. The implementation process of optimal sensing settings to achieve maximal SU spectral efficiency for the novel ST-CSS with hard decision fusion is presented in Algorithm 1. The initial values $\mu_{1}$ and the accuracy tolerance ζ f root are the two basic factors that affect the complexity and delay for this algorithm according to the convergence theorem of Newton–Raphson. The value of the former is related to ⌊K/2⌋ in the expression of Equation (63). Obviously, the former lies in the interzone (0.5, 1) because the target cooperative detection probability ${\hat{Q}}_{d}^{th}$ is selected to be 90%. Concerning the latter, the accuracy tolerance of the root has been chosen as 0.01 from the aspect of root calculation accuracy and convergence rate. After that, the maximal output spectral efficiency under different hard counting fusion rules can be obtained based on the comparison of their corresponding reporting order of all the SUs.
**Algorithm 1.** Optimal sensing settings that maximize the spectral efficiency.**Input:**
$\mu_{1}$ {$\mu_{1}$ is the initial guess of the root of $\Gamma ({\hat{p}}_{d,i})=0$ };   ζ {ζ the accuracy tolerance of the root};   $\Delta t_{i}$ {$\Delta t_{i}$ is the equivalent compressed sensing time for *i-*th SU, i=1,2,...,K};   $\gamma_{s}$ {$\gamma_{s}$ is the sensing SNR received at *i-*th SU from PU, s=1,2,...,K};**For**
n=1 , ⌊K/2⌋ , K
**Initiation:**
m←1, $\left\{\gamma_{i}|i=1,2...,K\right\}$; **While**: $\left|\;\Gamma (\mu_{m})\;\right|>\zeta$
  **do**   $\mu_{m+1}=\mu_{m}-\Gamma (\mu_{m})/\Gamma '(\mu_{m})$;   m←m+1; **End While**

${\hat{p}}_{d,i}^{n}\leftarrow \mu_{m}$
 **For:**
i=1,2...,K
  $\left\{\gamma_{s}\right\}=\left\{\gamma_{s}\right\}/\left\{\gamma_{i}\right\}$;  $\gamma_{i}\in \left\{\gamma_{s}\right\}$;  $\Delta t_{i}=\tau +(i-1)t_{r}$; Calculate ${\hat{p}}_{f,i}^{n}$ using Equation (55), $\hat{Q}{\;}_{f}^{n}$ using Equation (56), ${\hat{C}}^{n}$ using Equation (58); **End for****End for**Compare ${\hat{C}}^{\;n}$ and select the maximum, and noting corresponding $\left\{\gamma_{i}|i=1,2...,K\right\}$;**Output:**
${\left({\hat{C}}^{n}\right)}_{max}$


## 5. Simulation Results

This section provides extensive computer simulations carried out jointly using STK and MATLAB to evaluate the performance of SU spectral efficiency for the proposed spectrum sensing scheme. For analysis, we have considered the number of cooperating SUs is K=4 unless otherwise stated. The prior probabilities of presence and absence of PU are set to be $p({ℋ}_{0})=0.8$ and $p({ℋ}_{1})=1-p({ℋ}_{0})=0.2$. We suppose that the number of samples collected by each SU is *N* = 1000, and compressed domain sampling frequency is *f_s_* = 10 KHz. The PU’s power is $\sigma_{s}^{2}=10$ dBmW, the noise power $\sigma_{n}^{2}=-10$ dBmW the channel gain between PU and *i-*th SU $h_{\;i}$ obeys the Rayleigh distribution with mean $\bar{h}=-10$ dB, the channel gain among SU *g* = 0 dB. For the novel CSS scheme, the frame duration is *T* = 200 ms, in which the single reporting duration *t_r_* = 1 ms when the information transmission rate from SU to FC is assumed to be 1 kbps. The chosen LEO height is *h* = 1000 km, and the feedback slot is approximately given as *t*_0_ = 10 ms taking space propagation delay and final decision-making delay into account.

**Figure 6:**
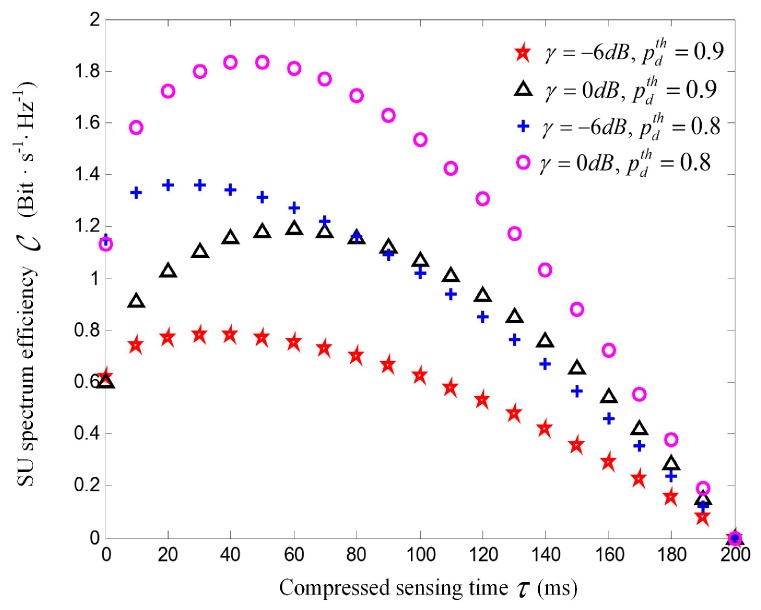
Spectral efficiency C versus sensing time τ under different levels of SNR and $p_{d}^{th}$.

Figure 6 shows SU spectrum utilization efficiency versus the compressed sensing time allocated to each SU. The simulation results validate that SU spectral efficiency C is the unimodal convex function with regard to compressed sensing time τ. There indeed exits one optimal point $\tau_{opt}$ standing in the curve that maximizes the SU spectral efficiency. We can see that the spectral efficiency increases as τ increases from 0 to $\tau_{opt}$, whereas decreases when τ is increased further. This is because little sensing time τ degrades sensing performance, while long sensing time τ results in short transmission duration. For a given SNR, C decreases with increased targeted detection probability. Particularly, for SNR = −6 dB, the maximal spectrum efficiency is 1.4 Bits·s^−1^·Hz^−1^ when target detection probability is 0.8, while it is only 0.8 Bits·s^−1^·Hz^−1^ and 0.5 Bits·s^−1^·Hz^−1^ when target detection probability is 0.9 and 0.95, respectively, which denotes that SU spectrum utilization decreases with debasement of the interference to PU. It can also be seen that optimal sensing time $\tau_{opt}$ increases with the increase of SNR values. Taking $p_{d}^{th}=0.9$ for example, when SNR = −6 dB, $\tau_{opt}=$ 30 *ms* to achieve the maximal spectral efficiency; when SNR = 0 dB, $\tau_{opt}=$ 60 *ms* to achieve the maximal spectral efficiency.

**Figure 7:**
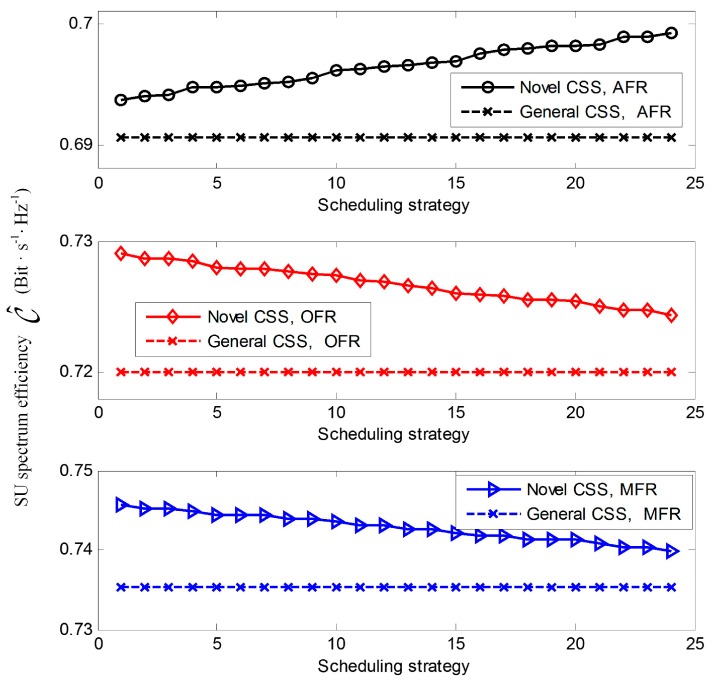
Spectral efficiency $\hat{C}$ versus scheduling strategy for the novel CSS scheme under fusion rules of AFR (and fusion rule), OFR (or fusion rule) and MFR (majority fusion rule), respectively.

In order to achieve a tradeoff between interference to PU and spectral efficiency, the target cooperative detection probability ${\hat{Q}}_{d}^{th}$ is set to be 0.9. In Figure 7, we plot SU spectral efficiency of the novel CSS with different reporting scheduling strategy, when the optimal sensing time is employed to cases where n is fixed at 1 (OFR), n is fixed at 2 (MFR), and n is fixed at 4 (AFR). The SNR $\gamma_{i}$ at the *i-*th SU is set to be {−20 dB, −16 dB, −12 dB, −8 dB}. The first scheduling strategy is the descending sort of received $\gamma_{i}$, while the last is the ascending sort of $\gamma_{i}$ among the total 24 cases of scheduling strategy. From Figure 7, it shows the novel CSS scheme outperforms the general CSS in the frequency reuse. In fact, the spectral efficiency of the novel CSS is closely related to scheduling strategy closely, while it has no effect on the general CSS. The optimal scheduling strategy for AFR is reporting the sensing results in the ascending sort of SNR, and reporting the sensing results in the descending sort of SNR can the unoccupied spectrum been fully utilized for OFR and MFR. This is consistent with the aforementioned theoretical proof.

**Figure 8:**
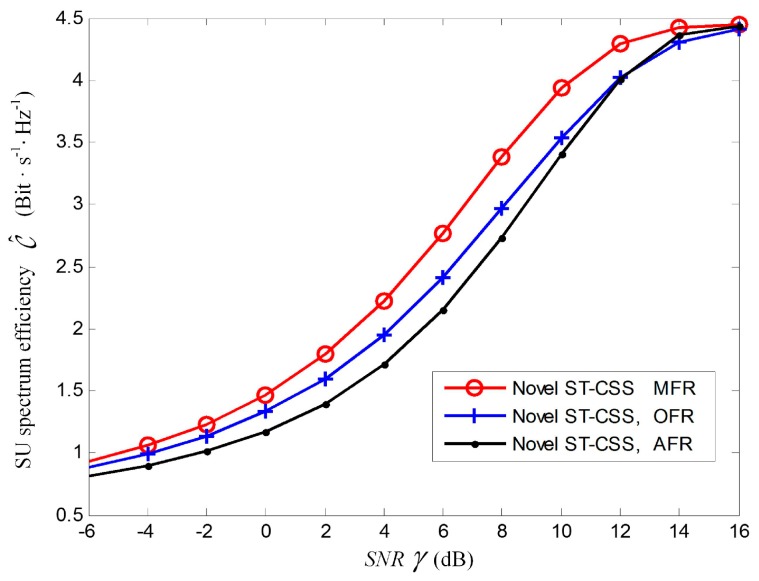
SU spectral efficiency $\hat{C}$ versus SNR γ for the Novel ST-CSS scheme under hard decision fusion rules of OFR, MFR and AFR, respectively.

Figure 8 compares SU spectral efficiency $\hat{C}$ of the proposed ST-CSS scheme versus sensing SNR with CSS based on general frame structure, when the algorithm of optimal sensing settings is employed to decision fusion rules of OFR, MFR, and AFR, respectively. It is assumed that M=5 and ${\hat{Q}}_{d}^{th}=0.9$. In Figure 8, it is obviously seen that the spectral efficiency enhances as SNR increases. Moreover, MFR exhibits the best performance of spectral efficiency than other two combining fusion rules for all SNR values. In lower SNR region of −6 dB~12 dB, OFR is sub-optimal, while AFR becomes sub-optimal when the value of SNR in higher region ranged from 10 dB~16 dB. In the SNR region of more than 16 dB, there is little difference in respect of SU spectrum usage for those three decision combing schemes. It is further concluded that MFR is the optimal rule which aims at maximizing the spectral efficiency with sufficient protection to PU.

**Figure 9:**
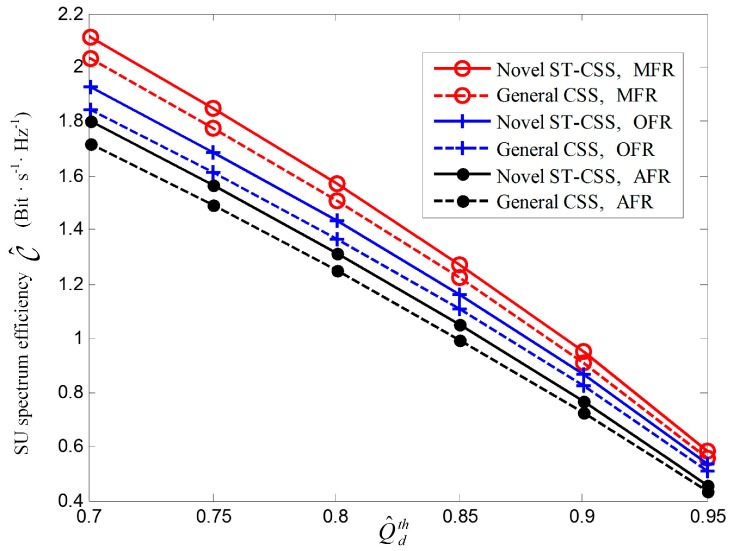
SU spectral efficiency $\hat{C}$ versus $\hat{Q}{\;}_{d}^{th}$ for the scheme of Novel ST-CSS and General CSS under decision fusion rules of OFR, MFR and AFR, respectively.

Figure 9 presents SU spectral efficiency versus the targeted cooperative detection probability $\hat{Q}{\;}_{d}^{th}$ for novel ST-CSS and general CSS with counting fusion schemes, in which the average SNR $\bar{\gamma }$ is −6 dB and the optimal sensing time is employed. As the dashed curves shown in Figure 9, the novel ST-CSS improves SU spectral efficiency compared with the general CSS. This is because in the framework of the novel ST-CSS scheme, individual SU reporting time can be used for other SU sensing. In this way, each SU gets a longer sensing duration without additional time overhead and the probability of false alarm can be reduced, which results in higher SU spectral efficiency. Additionally, aggregating the sensing results at different time mini-slots is expected to actualize a time diversity gain in spectrum utilization, which contributes to SU spectral efficiency. It is also can be seen from Figure 9 that SU spectral efficiency increases with the decreasing protection to PU. A relaxing constrained on $\hat{Q}{\;}_{d}^{th}$ can identify more spectrum opportunities, especially when $\hat{Q}{\;}_{d}^{th}$ becomes larger. For example, the SU spectral efficiency for novel ST-CSS with MFR improves approximately 14% when $\hat{Q}{\;}_{d}^{th}$ decreases from 0.75 to 0.5. However, it enhances 63% when ${\hat{Q}}_{d}^{th}$ decreases from 0.95 to 0.9. Therefore, the novel ST-CSS with MFR achieves best spectrum reuse with the same protection constraint.

**Figure 10:**
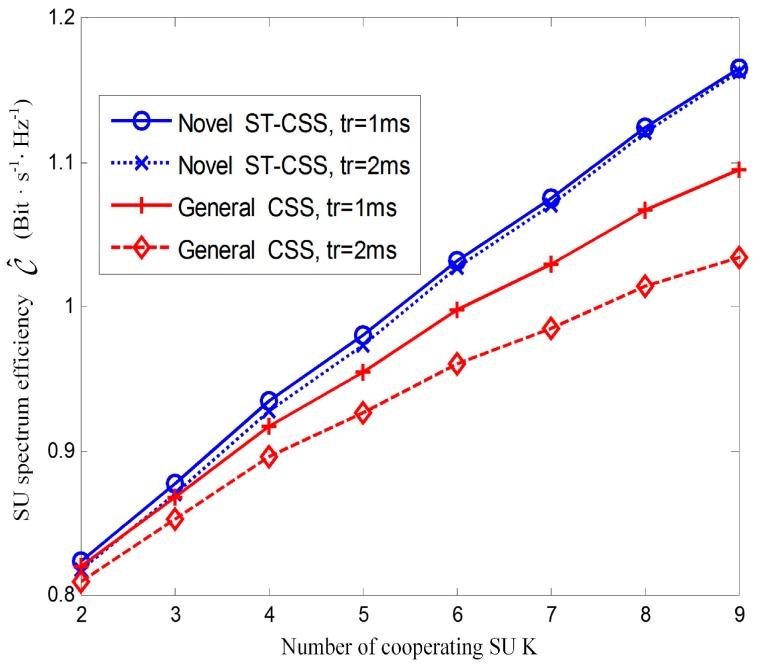
Spectral efficiency versus number of cooperating SU for the novel ST-CSS scheme and the general CSS scheme under different values of reporting time.

Figure 10 shows SU spectral efficiency versus the number of cooperating SU for the proposed ST-CSS scheme and the general CSS scheme under different values of reporting time. The optimal compressed sensing time and the optimal fusion rule are adopted in this simulation. The spectral efficiency relies on both reporting time $t_{r}$ and number of secondary user K. For K=9, the spectral efficiency of the novel ST-CSS scheme and the general CSS scheme are approximately 1.180 and 1.088 when *t_r_* = 1 ms, and approximately 1.178 and 1.032 when *t_r_* = 2 ms. In the case of K=4, the spectral efficiency of the two schemes are approximately 0.940 and 0.915 when *t_r_* = 1 ms, and approximately 0.934 and 0.896 when *t_r_* = 2 ms, respectively. Thus, the novel ST-CSS scheme can achieve a significant improvement on spectral efficiency than that of the general CSS scheme. Additionally, it is clear that more time spending for reporting sensing results to FC predicts lower spectral efficiency, especially for the general CSS. This is because in the conventional CSS structure, there is less time for transmission when allocates more time for reporting for the fixed frame length. However, in the framework of the novel ST-CSS scheme, the sensing performance can be further enhanced with extended equivalent sensing duration, which can compensate the loss of average throughput resulted from the less transmission time. It is further summarized that the proposed ST-CSS scheme is less sensitive to reporting time and significantly improves SU spectral efficiency.

**Figure 11:**
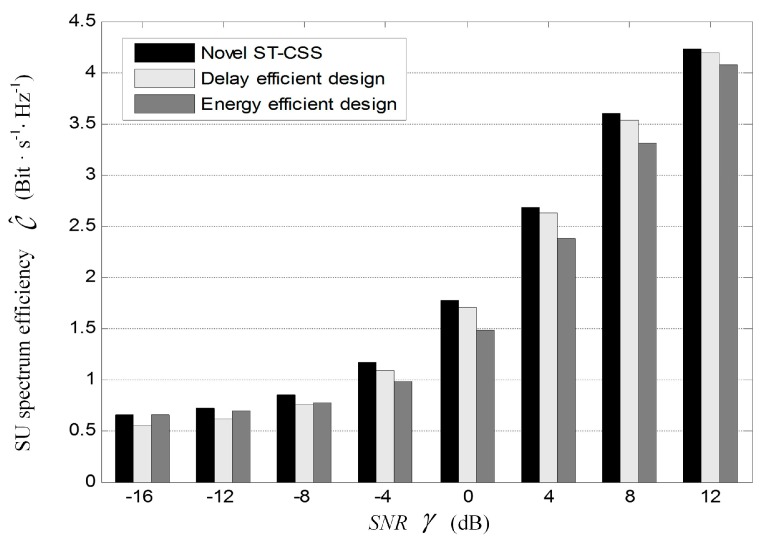
Comparisons of SU spectral efficiency among the schemes of the proposed ST-CSS, the transmission delay efficient design and the energy efficient design.

Figure 11 contrasts spectrum utilization efficiency of the proposed ST-CSS with the schemes of energy efficient design in [34] and transmission delay efficient design in [35], which satisfies the interference constraint on PU (i.e., $\hat{Q}{\;}_{d}^{th}=0.9$). As is depicted in Figure 11, our proposed ST-CSS scheme aiming at maximizing spectral efficiency in Equation (58) provides the highest spectral efficiency, in all values of SNR, while energy efficient design is sub-optimal in lower SNR and delay efficient design becomes sub-optimal in higher SNR. Numerically, the proposed solution improves 1% to 21% and 1% to 19% more spectral efficiency than delay efficient design and energy efficient design in all values of SNR, and the degrees of improvement of the spectral efficiency is determined by SNR. This is because the goal of transmission delay efficient design is to design the sensing parameters that can minimize the SU transmission delay in the scenario of delay sensitive applications, while the goal of energy efficient design is to design the sensing parameters that can maximize the energy efficiency in energy limited CR network with the same fixed frame length. Furthermore, the complexity of those three schemes are equivalent, because they differ only from optimization target in corresponding scenario with the similar main steps as Algorithm 1 presents. Specially, our proposed scheme is more suitable to overcome the problem of spectrum scarcity.

**Figure 12:**
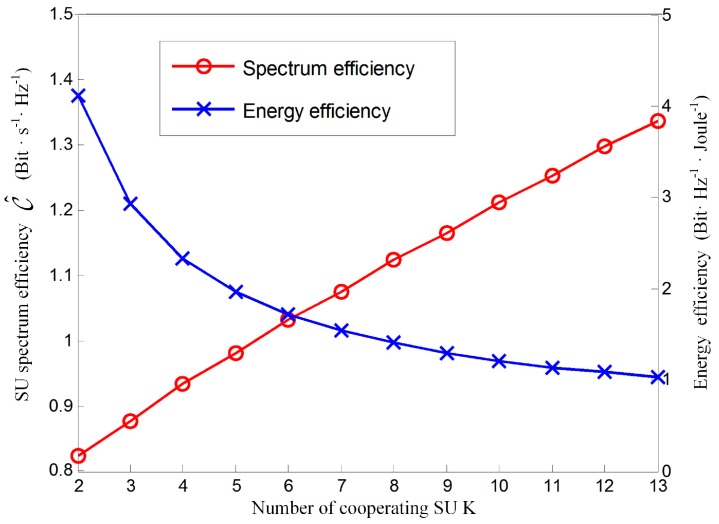
SU spectral efficiency and energy efficiency versus the number of cooperating SU for the novel ST-CSS scheme.

In order to evaluate the energy consumption of the proposed ST-CSS method in scenario of energy limited CR system, Figure 12 illustrates the SU energy efficiency and spectral efficiency of the novel ST-CSS scheme versus the number of cooperating SU synchronously. In this simulation, the energy efficient design in [34] is utilized to obtain the energy efficiency. Meanwhile, the optimal sensing time and the optimal combining rule are employed to obtain the SU spectral efficiency. It can be observed that the SU spectral efficiency enhances when K increases as a result of the improved cooperative sensing performance. However, energy efficiency will decrease as K increases, because more energy will be consumed to implement cooperative sensing and report sensing results. Though the more cooperative secondary sensor nodes in novel ST-CSS scheme can afford better spectral efficiency, it would shorten the CR networks lifetime due to increasing energy consumption.

## 6. Conclusions and Future Work

In this paper, we consider the spectrum scarcity issue in the scenario of LEO-based mobile satellite systems and investigate the joint optimization of sensing time and hard counting fusion schemes to maximize the spectral efficiency subject to sufficient protection to PU. We formulate the wideband compressed detection model based on the allocation of L/S band for mobile satellite services promulgated by ITU-RR. It is proven that spectral efficiency is the unimodal function of sensing time, and there indeed exists one optimal sensing time that maximizes the spectral efficiency. In order to make full use of the reporting slot, we propose the novel wideband cooperative spectrum sensing (CSS) framework considering the particular characteristics of LEO systems in which each SU reporting duration has been utilized for its following SUs’ sensing. On this basis, we derive the optimal reporting scheduling strategy of hard counting fusion rules with the consideration of different sensing SNR at SU in the novel CSS framework. Furthermore, the novel ST-CSS scheme is also presented for time-varying sensing environment to synchronously achieve time and space diversity gain. Analytical and simulation results provided the explicit impacts of target detection probability, number of cooperating SU, reporting time and the counting fusion rules on the spectral efficiency. The proposed scheme with optimal sensing settings achieved higher spectral efficiency compared to general CSS scheme and it is indeed needed in the scenario of spectrum scarce applications for LEO-based MSSs.

The methods in this paper can be used in improving cognitive satellite systems in the aspect of spectral efficiency. However, some practical conditions, such as the imperfect reporting link, should be considered to make our work stronger. In addition, the tradeoff between spectral efficiency and latency will be another future work for the sake of PU’s random arrival and departure within one frame length in the LEO-based mobile satellite systems with high traffic.

## Figures and Tables

**Table 1:** L/S band allocation for MSSs by ITU-R.

L/S Band	Uplink (MHz)	Downlink (MHz)	Bandwidth (MHz)	Area	Operational Systems
L band (1–2 GHz)	1610.0–1626.5	2483.5–2500.0	16.5	Global	Iridium, Globalstar
	1626.5–1660.5	1525.0–1559.0	34.0	Global	Inmarsat, MSAT, SkyTerra, ACeS, Thuraya
	1668.0–1675.0	1518.0–1525.0	7.0	Global	AlphaSat I-XL
S band (2–4 GHz)	1980–2010	2170–2200	30	Global	TerreStar, ICO
	2670–2690	2500–2520	20	Region III	Insat, N-STAR
	2655–2670	2520–2535	15	Region III	N-STAR

## References

[1] Chini P., Giambene G., Kota S. (2010). A survey on mobile satellite systems. Int. J. Satell. Commun. Netw..

[2] Alagoz F., Gur G. (2011). Energy efficiency and satellite networking: A holistic overview. Proc. IEEE.

[3] Celandroni N., Ferro E., Gotta A. (2013). A survey of architectures and scenarios in satellite-based wireless sensor networks: System design aspects. Int. J. Satell. Commun. Netw..

[4] Shree K.S., Symeon C., Bjorn O. (2016). In-line interference mitigation techniques for spectral coexistence of GEO and NGEO satellites. Int. J. Satell. Commun. Netw..

[5] Albuquerque J. Key note speech-what is going on in commercial satellite communications. Proceedings of the International Workshop on Satellite and Space Communications 2007 (IWSSC’07).

[6] Arcidiacono A., Finocchiaro D., Grazzini S. Broadband mobile satellite services: The Ku-band Revolution. Proceedings of the 2006 Tyrrhenian International Workshop on Digital Communications (TIWDC'06).

[7] ITU-R (2012). Radio Regulations Articles.

[8] Mitola J., Maguire G.Q. (1999). Cognitive Radio: Making software radios more personal. IEEE Pers. Commun..

[9] Haykin S. (2005). Cognitive radio: Brain-empowered wireless communications. IEEE J. Sel. Areas Commun..

[10] Zou Y., Yao Y., Zheng B. (2012). Cooperative relay techniques for cognitive radio systems: Spectrum sensing and secondary user transmissions. IEEE Commun. Mag..

[11] Liang Y., Chen K.C., Li G.Y., Mahonen P. (2011). Cognitive radio networking and communications: An overview. IEEE Trans. Veh. Technol..

[12] Biglieri E. An overview of cognitive radio for satellite communications. Proceedings of the 2012 IEEE First AESS European Conference on Satellite Telecommunications (ESTEL).

[13] Hoyhtya M., Kyrolainen J., Hulkkonen A., Ylitalo J., Roivainen A. Application of cognitive radio techniques to satellite communication. Proceedings of the 2012 IEEE International Symposium on Dynamic Spectrum Access Networks (DYSPAN).

[14] Dimc F., Baldini G., Kandeepan S. (2015). Experimental detection of mobile satellite transmissions with cyclostationary features. Int. J. Satell. Commun. Netw..

[15] Clark M., Psounis K. (2017). Equal Interference Power Allocation for Efficient Shared Spectrum Resource Scheduling. IEEE Trans. Wirel. Commun. Netw..

[16] Hoyhtya M. (2013). Secondary terrestrial use of broadcasting satellite services below 3 GHz. Int. J. Wirel. Mob. Netw..

[17] Khan B.M., Mustaqim M., Khawaja B.A., ShabeehUlHusnain S. (2016). Spectrum sensing in satellite cognitive radios: Blind signal detection technique. Wiley Microw. Opt. Technol. Lett..

[18] Maleki S., Chatzinotas S., Evans B. (2015). Cognitive spectrum utilization in Ka band multibeam satellite communications. IEEE Commun. Mag..

[19] Havary-Nassab V., Hassan S., Valaee S. Compressive detection for wide-band spectrum sensing. Proceedings of the IEEE International Conference on Acoustics Speech and Signal Processing.

[20] Donoho D. (2006). Compressed sensing. IEEE Trans. Inf. Theory.

[21] Bazerque J.A., Giannakis G.B. (2010). Distributed spectrum sensing for cognitive radio networks by exploiting sparsity. IEEE Trans. Signal Process..

[22] Tian Z., Giannakis G.B. Compressed sensing for wideband cognitive radios. Proceedings of the IEEE International Conference on Acoustics, Speech, and Signal Processing (ICASSP).

[23] Duarte M.F., Eldar Y.C. (2011). Structured Compressed Sensing: From Theory to Applications. IEEE Trans. Signal Process..

[24] Leus G., Ariananda D.D. (2011). Power spectrum blind sampling. IEEE Signal Process. Lett..

[25] Mark A.D., Michael B.W., Richard G.B. (2007). Detection and Estimation with Compressive Measurements.

[26] Jia M., Liu X., Gu X.M. (2015). Joint cooperative spectrum sensing and channel selection optimization for satellite communication systems based on cognitive radio. Int. J. Satell. Commun. Netw..

[27] Wang Y.L., Zhang G.X., Bian D.M., Gou L., Zhang W. (2014). Collaborative Wideband Compressed Signal Detection in Interplanetary Internet. Frequenz.

[28] Yucek T., Arslan H. (2009). A survey of spectrum sensing algorithms for cognitive radio applications. IEEE Commun. Surv. Tutor..

[29] Zhang W., Guo Y., Liu H., Chen Y., Wang Z., Mitola J. (2015). Distributed consensus-based weight design for cooperative spectrum sensing. IEEE Trans. Parallel Distrib. Syst..

[30] Ebrahimzadeh A., Najimi M., Andargoli S., Fallahi A. (2015). Sensor selection and optimal energy detection threshold for efficient cooperative spectrum sensing. IEEE Trans. Veh. Technol..

[31] Liu X., Jia M., Gu X.M., Tan X.Z. (2013). Optimal periodic cooperative spectrum sensing based on weight fusion in cognitive radio networks. Sensors.

[32] Zhang D.Y., Chen Z.G., Ren J., Zhang N., Award M.K., Zhou H.B., Shen X.M. (2017). Energy Harvesting-Aided Spectrum Sensing and Data Transmission in Heterogeneous Cognitive Radio Sensor Network. IEEE Trans. Veh. Technol. Netw..

[33] Peh E., Liang Y.C., Guan Y.L. (2009). Optimization of cooperative spectrum sensing in cognitive radio networks: A sensing-throughput tradeoff view. IEEE Trans. Veh. Technol..

[34] Shi Z., Teh K.C., Li K.H. (2013). Energy-efficient joint design of sensing and transmission duration for protection of primary user in cognitive radio systems. IEEE Commun. Lett..

[35] Hu H., Zhang H., Yu H. (2014). Efficient Spectrum Sensing with Minimum Transmission Delay in Cognitive Radio Networks. Mobile Netw. Appl..

[36] Lee W.-Y., Akyildiz I.F. (2008). Optimal Spectrum Sensing Framework for Cognitive Radio Networks. IEEE Trans. Wirel. Commun..

[37] Maleki S., Chepuri S.P., Leus G. (2013). Optimization of hard fusion based spectrum sensing for energy constrained cognitive radio networks. Phys. Commun..

[38] Kay S.M. (1998). Fundamentals of Statistical Signal Processing.

[39] Alaa A.M., Nasr O.A. A globally optimal Neyman-Pearson test for hard decisions fusion in cooperative spectrum sensing. Proceedings of the Computing, Networking and Communications (ICNC).

[40] Tang W.C., Shakir M.Z., Imran M.A. (2016). Spectral and energy efficient cognitive radio-aided heterogeneous cellular network with uplink power adaptation. Wirel. Commun. Mob. Comput..

[41] Beaulieu N.C., Chen Y. (2010). Improved energy detector for cognitive radios with randomly arriving or departing primary users. IEEE Signal Process. Lett..

